# Supplementary material for: Clinical impact of pharmacogenetic risk variants in a large chinese cohort
Source: Nat Commun. 2025 Jul 9;16:6344. doi: 10.1038/s41467-025-61644-x (PMC12241563; doi:10.1038/s41467-025-61644-x)

Supplementary Table 1: List of clinically relevant PGx variants

Gene	Allele	Array Type	Data source	Allele Functional Status^	Activity Score*	TPMI_AF	Sensitivity[$]	Specificity[$]
ABCG2	c.421 C>A	TPM/TPM2	Genotype	Decreased function	N/A	31.7%	99.9%	100.0%
CYP2B6	rs3745274[#]	TPM/TPM2	Genotype	Decreased function	N/A	19.1%	99.8%	100.0%
CYP2C9	*3	TPM/TPM2	Genotype	Decreased function	0.5	3.2%	100.0%	100.0%
	*16	TPM/TPM2	Genotype	Decreased function	0.5	0.5%	100.0%	100.0%
CYP2C19	*2	TPM/TPM2	Genotype	No function	N/A	32.3%	100.0%	100.0%
	*3	TPM/TPM2	Genotype	No function	N/A	5.2%	100.0%	100.0%
	*6	TPM/TPM2	Genotype	No function	N/A	0.2%	100.0%	100.0%
	*17	TPM/TPM2	Imputation	Increased function	N/A	0.5%	100.0%	100.0%
CYP3A5	*1	TPM/TPM2	Genotype	Normal function		28.6%	100.0%	100.0%
CYP4F2	*3	TPM/TPM2	Genotype	Decreased function		23.6%	100.0%	100.0%
G6PD	Canton/Taiwan-Hakka	TPM/TPM2	Imputation/Genotype	Deficient function	II	1.41%	100.0%	100.0%
	Chinese-3/taipei	TPM/TPM2	Genotype	Deficient function	III	0.20%	100.0%	100.0%
	Chinese-5	TPM/TPM2	Genotype	Deficient function	II	0.25%	100.0%	100.0%
	Gaohe	TPM/TPM2	Genotype	Deficient function	II	0.17%	100.0%	100.0%
	Kaiping	TPM/TPM2	Genotype	Deficient function	II	0.61%	100.0%	100.0%
	QuingYan	TPM/TPM2	Genotype	Deficient function	III	0.17%	100.0%	100.0%
HLA-A	*31:01	TPM/TPM2	HiBag	Risk		2.3%	100.0%	100.0%
HLA-B	*15:02	TPM/TPM2	HiBag	Risk		4.2%	100.0%	99.9%
	*57:01	TPM/TPM2	HiBag	Risk		0.2%	100.0%	100.0%
	*58:01	TPM/TPM2	HiBag	Risk		10.6%	100.0%	100.0%
HLA-DQA1	*02:01	TPM/TPM2	HiBag	Risk		2.4%	100.0%	100.0%
HLA-DRB1	*07:01	TPM/TPM2	HiBag	Risk		2.3%	96.3%	100.0%
IFNL3	rs12979860	TPM/TPM2	Genotype	Unfavorable response		5.8%	99.4%	100.0%
MTRNR1	m.1095T>C	TPM/TPM2	Genotype	Risk		0.2%	100.0%	100.0%
	m.1555A>G	TPM/TPM2	Genotype	Risk		0.2%	100.0%	100.0%
NAT2	*5	TPM/TPM2	Genotype	Decreased Function		3.9%	100.0%	100.0%
	*6	TPM/TPM2	Genotype	Decreased Function		26.5%	100.0%	100.0%
	*7	TPM/TPM2	Genotype	Decreased Function		16.1%	100.0%	100.0%
NUDT15	*2/*3	TPM/TPM2	Imputation/Genotype	No Function		11.0%	99.7%	100.0%
SLCO1B1	*5/*15	TPM/TPM2	Genotype	No function		11.2%	100.0%	100.0%
TPMT	*3C	TPM/TPM2	Genotype	No Function		1.4%	100.0%	100.0%
UGT1A1	*6	TPM/TPM2	Genotype	Decreased Function		15.3%	100.0%	100.0%
	*27	TPM/TPM2	Genotype	Decreased Function		2.1%	100.0%	100.0%
	rs887829	TPM/TPM2	Genotype	Decreased Function		12.1%	100.0%	100.0%
VKORC1	-1639G>A	TPM/TPM2	Genotype	Require warfarin dose reduction		89.3%	100.0%	100.0%

^ PharmGKB Gene-specific Information Tables https://www.pharmgkb.org/page/pgxGeneRef
PharmVar 5.0.1 https://www.pharmvar.org/

* PharmGKB Gene-specific Information Tables https://www.pharmgkb.org/page/pgxGeneRef

[$] Evaluated the condordance of genotype and imputation calls with NGS or Sanger sequencing data

[#] Shared by decreased or no functional CYP2B6 alleles, including *6, *7, *9, *13, *19, *20, *26, *34, *36, *37, *38, *39, *40, *41, *42, *43

Supplementary Table 2: Distribution of PGx phenotype, actionable phenotypes, and risk drug exposure

Category	Gene	Phenotype	Actionable Phenotype	Case number	Phenotype Freq		Risk drug	Risk drug exposure freq in each phenotype	Individuals encountered following high-risk drugs in each phenotype group													
									Efavirenz	Sertraline												
Phase 1 Metabolic Enzymes	CYP2B6	Total		486,956			13,118	2.7%	258	4,786												
		EM		318,276	65.4%	318879	8,539	2.7%	179	3,086												
		IM	v	149,998	30.8%	168,077	4,082	2.7%	73	1,507												
		PM	v	18,079	3.7%		487	2.7%	6	189												
		Indetermined		603	0.1%		10	1.7%	0	4												
									Celecoxib	Flurbiprofen	Fluvastatin	Ibuprofen	Meloxicam	Nateglinide	Phenytoin	Piroxicam	Tenoxicam	Warfarin				
	CYP2C9	Total		486,956			92,103	18.9%	50,200	28,989	4,219	6,681	10,174	227	670	13,933	23	6,113				
		EM		451,020	92.6%	451,393	85,301	18.9%	46,570	26,807	3,903	6,135	9,447	210	623	12,841	23	5,665				
		IM	v	35,032	7.2%	35,563	6,639	19.0%	3,556	2,122	302	529	715	17	46	1,061	-	439				
		PM	v	531	0.1%		97	18.3%	52	37	9	12	7		1	17		7				
		Indetermined		373	0.1%		66	17.7%	22	23	5	5	5			14		2				
									Abrocitinib	Brivaracetam	Citalopram	Clobazam	Clopidogrel	Dexlansoprazol	Doxepin	Escitalopra	Imipramin	Lansoprazol	Omeprazole	Pantoprazol	Sertraline	Voriconazole
		Total		486,956			116,120	23.8%	11	28	6,942	1,283	33,664	19,147	6,773	6,845	9,636	44,378	33,407	28,138	4,786	343
	CYP2C19	UM	v	17	0.0%	187,877		0.0%														
		RM	v	3,183	0.7%	299,079	751	23.6%			51	6	229	122	44	51	46	284	210	185	30	3
		EM		187,095	38.4%		44,657	23.9%	4	13	2,714	478	12,872	7,387	2,588	2,677	3,802	17,063	12,807	10,889	1,821	133
		IM	v	226,301	46.5%		53,742	23.7%	5	12	3,196	611	15,667	8,866	3,157	3,146	4,393	20,613	15,453	12,967	2,222	159
		PM	v	69,578	14.3%		16,787	24.1%	2	3	964	188	4,850	2,738	974	954	1,374	6,342	4,891	4,057	706	48
		na		782	0.2%		183	23.4%			17		46	34	10	17	21	76	46	40	7	
									Tacrolimus													
	CYP3A5	Total		486,956			3,299	0.7%	3,299													
		PM		248,453	51.0%	248,578	1,668	0.7%	1,668													
		IM	v	198,492	40.8%	238,378	1,341	0.7%	1,341													
		EM	v	39,886	8.2%		289	0.7%	289													
		Indetermined		125	0.0%		1	0.8%	1													
									Warfarin													
	CYP4F2	Total		486,956			6,113	1.3%	6,113													
		EM		284,173	58.4%	285,017	3,578	1.3%	3,578													
		IM	v	174,577	35.8%	201,939	2,174	1.2%	2,174													
		PM	v	27,362	5.6%		352	1.3%	352													
		Indetermined		844	0.2%		9	1.1%	9													
									Isoniazid	Sulfamethoxazole and Trimethoprim	Sulfasalazine											
Phase 2 Metabolic Enzymes	NAT2	Total		486,956			19,565	4.0%	2,918	7,699	9,777											
		EM		139,510	28.6%	382,014	5,597	4.0%	854	2,172	2,803											
		IM		241,822	49.7%	104,942	9,727	4.0%	1,415	3,844	4,876											
		PM	v	104,942	21.5%		4,205	4.0%	646	1,666	2,082											
		Indetermined		682	0.1%		36	5.3%	3	17	16											
									Azathioprine	Mercaptopurine												
	NUDT15	Total		486,956			8,523	1.8%	8,486	37												
		EM		384,231	78.9%	386,050	6,820	1.8%	6,789	31												
		IM	v	95,046	19.5%	100,906	1,595	1.7%	1,590	5												
		PM	v	5,860	1.2%		70	1.2%	70													
		Indetermined		1,819	0.4%		38	2.1%	37	1												
									Azathioprine	Mercaptopurine												
	TPMT	Total		486,956			8,523	1.8%	8,486	37												
		EM		473,144	97.2%	473,360	8,279	1.7%	8,243	36												
		IM	v	13,479	2.8%	13,596	241	1.8%	240	1												
		PM	v	117	0.0%		1	0.9%	1													
		Indetermined		216	0.0%		2	0.9%	2													
									Atazanavir	Irinotecan	Nilotinib	Pazopanib										
	UGT1A1	Total		486,956			845	0.2%	56	596	73	120										
		EM		255,461	52.5%	258,998	447	0.2%	31	324	38	54										
		IM	v	176,543	36.3%	227,958	304	0.2%	19	208	28	49										
		PM	v	51,415	10.6%		84	0.2%	6	56	6	16										
		Indetermined		3,537	0.7%		10	0.3%	-	8	1	1										
									Rosuvastatin													
Transprote	ABCG2	Total		486,956			46,617	9.6%	49,032													
		Normal		227,345	46.7%	437,794	21,588	9.5%	22,702													
		Decreased		210,293	43.2%	49,162	20,314	9.7%	21,384													
		Poor	v	49,162	10.1%		4,703	9.6%	4,932													
		Indetermined		156	0.0%		12	7.7%	14													
									Atorvastatin	Fluvastatin	Lovastatin	Pitavastatin	Pravastatin	Rosuvastatin	Simvastatin							
	SLCO1B1	Total		486,956			127,197	26.1%	73,078	4,219	4,154	32,334	7,786	49,032	11,558							
		Normal		383,660	78.8%	383,711	100,390	26.2%	57,718	3,314	3,248	25,416	6,140	38,557	9,000							
		Decreased	v	97,031	19.9%	103,245	25,163	25.9%	14,440	847	839	6,498	1,540	9,817	2,391							

		**Poor**	**v**	6,214	1.3%		1,637	26.3%	915	58	67	419	106	655	166
		**Indetermined**		51	0.0%		7	13.7%	5	-	-	1	-	3	1
									**Carbamazepine**						
**HLA**	**HLA-A*3101**	**Total**		486,956			1,319	0.3%	1,319						
		**Negative**		467,890	96.1%		1,278	0.3%	1,278						
		**Positive**	**v**	19,066	3.9%		41	0.2%	41						
									**Carbamazepin**	**Oxcarbazepine**	**Phenytoin**				
	**HLA-B*1502**	**Total**		486,956			3,961	0.8%	1,319	2,229	670				
		**Negative**		446,907	91.8%		3,675	0.8%	1,247	2,056	616				
		**Positive**	**v**	40,049	8.2%		286	0.7%	72	173	54				
									**Abacavir**						
	**HLA-B*5701**	**Total**		486,956			692	0.1%	692						
		**Negative**		485,071	99.6%		691	0.1%	691						
		**Positive**	**v**	1,885	0.4%		1	0.1%	1						
									**Allopurinol**						
	**HLA-B*5801**	**Total**		486,956			5,251	1.1%	5,251						
		**Negative**		389,717	80.0%		4,373	1.1%	4,373						
		**Positive**	**v**	97,239	20.0%		878	0.9%	878						
									**Lapatinib**						
	**DRB1*0701**	**Total**		486,956			27	0.0%	27						
		**Negative**		464,494	95.4%		27	0.0%	27						
		**Positive**	**v**	22,462	4.6%			0.0%							
									**Lapatinib**						
	**DQA1*0201**	**Total**		486,956			27	0.0%	27						
		**Negative**		464,545	95.4%		27	0.0%	27						
		**Positive**	**v**	22,411	4.6%			0.0%							
									**Dapsone**	**Nitrofurantoin**	**Rasburicase**				
**Others**	**G6PD**	**Total**		486,956			144	0.0%	108	5	31				
		**normal**		446,940	91.8%	466,603	129	0.0%	96	4	29				
		**variable**	**v**	14,095	2.9%	20,353	6	0.0%	5	1					
		**deficient**	**v**	6,258	1.3%		3	0.0%	2		1				
		**Indetermined**		19,663	4.0%		6	0.0%	5		1				
									**Warfarin**						
	**VKORC1**	**Total**		486,956			6,113	1.3%	6,113						
		**0**	**v**	388,233	79.7%	5,796	4,803	1.2%	4,803						
		**1**	**v**	92,927	19.1%	481,160	1,237	1.3%	1,237						
		**2**		5,676	1.2%		70	1.2%	70						
		**Indetermined**		120	0.0%		3	2.5%	3						
									**Peginterferon alfa-2a**	**Peginterferon alfa-2b**	**Ribavirin**				
	**IFNL3**	**Total**		486,956			4,255	0.9%	576	218	1,873				
		**Favored**		431,219	88.6%	431,571	3,692	0.9%	504	181	1,591				
		**Unfavored**	**v**	55,385	11.4%	55,385	562	1.0%	72	37	282				
		**Indetermined**		352	0.1%		1	0.3%							
									**Amikacin**	**Gentamicin**	**Kanamycin**	**Paromomycin**	**Streptomycin**	**Tobramycin**	
	**MT-RNR1**	**Total**		486,956			27,595	5.7%	635	23,953	5	15	59	4,190	
		**Normal**		484,521	99.5%	485,059	27,441	5.7%	631	23,816	5	15	59	4,168	
		**Risk**	**v**	1,897	0.4%	1,897	121	6.4%	3	109				16	
		**Indetermined**		538	0.1%		33	6.1%	1	28				6	

Supplementary Table 3: The distribution of TPMI participants exposed to high-risk PGx drugs

Number of drug prescibed per person	Case number	Accumulated count	Accumulated %
0 (unexposed)	249,796		
1	98,724	237,160	48.7%
2	60,111	138,436	28.4%
3	33,926	78,325	16.1%
4	19,251	44,399	9.1%
5	10,596	25,148	5.2%
6	6,114	14,552	3.0%
7	3,550	8,438	1.7%
8	2,124	4,888	1.0%
9	1,133	2,764	0.6%
10	673	1,631	0.3%
11	440	958	0.2%
12	242	518	0.1%
13	132	276	0.1%
14	78	144	0.0%
15	42	66	0.0%
16	14	24	0.0%
17	9	10	0.0%
18	1	1	0.0%
**SUM**	**486,956**		

Supplementary Table 4: The demography of clopidogrel-related MACE cohort

**Clopidogrel Users**	**no MACE**		**MACE**		**OR (95% CI)**	**P value**
**No. of subjects**	24578		3477			
**Male**	8208	33.4%	1179	33.9%	0.98 (0.91-1.06)	5.49E-01
**Age (years, mean±SD)**	72.0±9.5		71.24±10.4			1.50E-01
**Comorbidity**						
**DM**	13700	55.7%	1910	54.9%	0.97 (0.9-1.04)	3.7E-01
**HC**	15803	64.3%	2219	63.8%	0.98 (0.91-1.06)	5.8E-01
**HTN**	11503	46.8%	1625	46.7%	1 (0.93-1.07)	9.1E-01
**CKD**	8852	36.0%	1209	34.8%	0.95 (0.88-1.02)	1.5E-01
**Concurrent medications**						
**aspirin**	2231	9.1%	324	9.3%	1.03 (0.91-1.16)	6.4E-01
**warfarin**	1253	5.1%	190	5.5%	1.08 (0.92-1.26)	3.6E-01
**PPI**	8670	35.3%	1420	40.8%	1.27 (1.18-1.37)	1.56E-10
**statin**	10061	40.9%	1458	41.9%	1.04 (0.97-1.12)	2.8E-01

Supplementary Table 5: Influence of CYP2C19 for incidence of MACE

Clopidogrel users with one or two CYP2C19 LoF vs with non-LoF

	Univariate analysis				Multivariate analysis			
MACE	OR	Lower95%CI	Upper95%CI	Pvalue	OR	Lower95%CI	Upper95%CI	Pvalue
Stroke	1.52	1.41	1.64	4.39E-27	1.53	1.42	1.65	2.96E-27
MI	4.01	3.18	5.05	9.13E-32	3.98	3.15	5.02	2.09E-31
UA	1.88	1.58	2.22	5.21E-13	1.88	1.58	2.23	7.24E-13
HF	1.37	1.22	1.55	1.46E-07	1.37	1.22	1.55	1.77E-07
TLR	1.29	1.13	1.47	1.33E-04	1.29	1.13	1.47	1.91E-04
CV death	1.28	0.85	1.92	2.37E-01	1.25	0.82	1.89	2.99E-01
MACE	1.40	1.18	1.65	7.90E-05	1.41	1.19	1.67	6.31E-05

Clopidogrel users with one CYP2C19 LoF vs with non-LoF

	Univariate analysis				Multivariate analysis			
MACE	OR	Lower95%CI	Upper95%CI	Pvalue	OR	Lower95%CI	Upper95%CI	Pvalue
Stroke	1.49	1.38	1.62	1.21E-22	1.50	1.39	1.63	6.76E-23
MI	3.95	3.12	5.01	7.51E-30	3.90	3.08	4.95	2.75E-29
UA	1.83	1.53	2.19	2.74E-11	1.84	1.53	2.20	3.38E-11
HF	1.34	1.18	1.51	5.42E-06	1.34	1.18	1.52	5.27E-06
TLR	1.28	1.11	1.46	5.93E-04	1.27	1.11	1.46	7.28E-04
CV death	1.24	0.81	1.91	3.20E-01	1.19	0.76	1.84	4.49E-01
MACE	1.38	1.16	1.65	2.94E-04	1.40	1.17	1.67	2.11E-04

Clopidogrel users with two CYP2C19 LoF vs with non-LoF

	Univariate analysis				Multivariate analysis			
MACE	OR	Lower95%CI	Upper95%CI	Pvalue	OR	Lower95%CI	Upper95%CI	Pvalue
Stroke	1.62	1.45	1.80	1.60E-18	1.62	1.46	1.81	2.05E-18
MI	4.18	3.17	5.53	6.21E-24	4.22	3.19	5.57	3.87E-24
UA	2.02	1.61	2.53	1.29E-09	2.02	1.60	2.54	1.90E-09
HF	1.50	1.27	1.77	1.65E-06	1.49	1.26	1.76	3.08E-06
TLR	1.35	1.12	1.63	1.72E-03	1.34	1.11	1.62	2.72E-03
CV death	1.39	0.79	2.46	2.56E-01	1.44	0.81	2.55	2.14E-01
MACE	1.45	1.15	1.84	1.72E-03	1.46	1.15	1.85	1.89E-03

Supplementary Table 6: Patient characteristics of azathioprine users in TPMI

Azathioprine Users	no ADE		ADE		OR (95% CI)	P value
**No. of subjects**	6948		1503			
**Male**	2140	30.8%	434	28.9%	0.91 (0.8-1.03)	1.40E-01
**Age (years, mean±SD)**	46.2±15		42.5±14.7			9.43E-02
**Comorbidity**						
**DM**	763	11.0%	170	11.3%	1.03 (0.86-1.23)	7.10E-01
**HC**	1329	19.1%	305	20.3%	1.08 (0.94-1.24)	3.00E-01
**HTN**	1304	18.8%	309	20.6%	1.12 (0.97-1.37)	1.10E-01
**IHD**	484	7.0%	115	7.7%	1.11 (0.9-1.37)	3.50E-01
**HF**	138	2.0%	34	2.3%	1.14 (0.76-1.67)	4.90E-01
**CVD**	292	4.2%	72	4.8%	1.15 (0.88-1.5)	3.10E-01
**Concurrent medications**						
**aspirin**	327	4.7%	61	4.1%	0.86 (0.65-1.14)	2.80E-01
**allopurinol**	67	1.0%	29	1.9%	2.02 (1.3-3.13)	1.37E-03
**corticosteroids**	5262	75.7%	1164	77.4%	1.1 (0.96-1.26)	1.60E-01
**methotrexate**	1030	14.8%	217	14.4%	0.97 (0.83-1.14)	7.00E-01

Supplementary Table 7: Influence of NUDT15 and TPMT for azathioprine discontinuation due to ADR

Azathioprine users with one or two NUDT15 LoF vs with non-LoF

	Univariate analysis				Multivariate analysis			
ADE	OR	Lower95%CI	Upper95%CI	Pvalue	OR	Lower95%CI	Upper95%CI	Pvalue
Leukopenia	1.34	1.17	1.53	0.0000	1.36	1.19	1.56	9.61E-06
Thrombocytoper	1.82	1.56	2.13	0.0000	1.85	1.58	2.17	4.47E-14
Hepatitis	1.17	0.95	1.43	0.1400	1.20	0.97	1.48	9.73E-02
Allergy	0.98	0.76	1.27	0.8820	0.99	0.76	1.29	9.19E-01
GI	1.16	0.59	2.26	0.6710	1.06	0.53	2.13	8.73E-01
HairLoss	1.29	0.79	2.11	0.3150	1.39	0.85	2.29	1.91E-01
ADR	1.21	0.65	2.26	0.5440	1.16	0.61	2.20	6.60E-01

Azathioprine users with one NUDT15 LoF vs with non-LoF

	Univariate analysis				Multivariate analysis			
ADE	OR	Lower95%CI	Upper95%CI	Pvalue	OR	Lower95%CI	Upper95%CI	Pvalue
Leukopenia	1.25	1.09	1.44	0.0013	1.28	1.11	1.48	5.44E-04
Thrombocytoper	1.69	1.44	1.99	0.0000	1.73	1.46	2.04	9.44E-11
Hepatitis	1.11	0.90	1.37	0.3460	1.14	0.91	1.42	2.45E-01
Allergy	0.95	0.73	1.24	0.7280	0.95	0.73	1.26	7.39E-01
GI	1.10	0.55	2.20	0.7950	0.99	0.48	2.05	9.82E-01
HairLoss	1.21	0.73	2.03	0.4570	1.32	0.79	2.21	2.97E-01
ADR	0.78	0.36	1.65	0.5110	0.80	0.37	1.71	5.61E-01

Azathioprine users with two NUDT15 LoF vs with non-LoF

	Univariate analysis				Multivariate analysis			
ADE	OR	Lower95%CI	Upper95%CI	Pvalue	OR	Lower95%CI	Upper95%CI	Pvalue
Leukopenia	4.24	2.63	6.84	2.95E-09	4.05	2.47	6.64	2.88E-08
Thrombocytoper	5.84	3.56	9.56	2.52E-12	5.89	3.51	9.90	1.96E-11
Hepatitis	2.68	1.40	5.14	3.03E-03	2.67	1.35	5.30	4.91E-03
Allergy	1.61	0.64	4.03	3.09E-01	1.76	0.70	4.41	2.29E-01
GI	2.54	0.34	18.72	3.62E-01	2.75	0.37	20.38	3.22E-01
HairLoss	2.98	0.72	12.43	1.33E-01	3.17	0.75	13.28	1.15E-01
ADR	11.93	4.58	31.07	3.85E-07	10.84	3.72	31.58	1.24E-05

Azathioprine users with one or two TPMT LoF vs with non-LoF

	Univariate analysis				Multivariate analysis			
ADE	OR	Lower95%CI	Upper95%CI	Pvalue	OR	Lower95%CI	Upper95%CI	Pvalue
Leukopenia	0.92	0.65	1.29	0.624	0.92	0.65	1.31	6.37E-01
Thrombocytoper	1.06	0.71	1.59	0.778	1.03	0.68	1.57	8.92E-01
Hepatitis	0.91	0.54	1.55	0.727	0.97	0.57	1.66	9.25E-01
Allergy	0.89	0.47	1.70	0.733	0.86	0.44	1.69	6.68E-01
GI	0.69	0.10	5.05	0.718	0.70	0.10	5.06	7.20E-01
HairLoss	0.79	0.19	3.23	0.743	0.84	0.20	3.43	8.07E-01
ADR	1.90	0.59	6.13	0.281	1.28	0.31	5.28	7.37E-01

Azathioprine users with one TPMT LoF vs with non-LoF

	Univariate analysis				Multivariate analysis			
ADE	OR	Lower95%CI	Upper95%CI	Pvalue	OR	Lower95%CI	Upper95%CI	Pvalue
Leukopenia	0.92	0.65	1.30	0.644	0.92	0.65	1.31	6.56E-01
Thrombocytoper	1.07	0.71	1.60	0.761	1.04	0.68	1.58	8.72E-01
Hepatitis	0.91	0.54	1.55	0.739	0.98	0.57	1.67	9.34E-01
Allergy	0.90	0.47	1.71	0.743	0.87	0.44	1.70	6.76E-01
GI	0.70	0.10	5.07	0.721	0.70	0.10	5.08	7.22E-01
HairLoss	0.79	0.19	3.24	0.748	0.84	0.21	3.45	8.11E-01
ADR	1.91	0.59	6.16	0.278	1.28	0.31	5.32	7.29E-01

Supplementary Table 8: Statin users with muscle complaints toward different types of statin

	SAM with how many statins						
**The type of statins precribed**	**0**	**1**	**2**	**3**	**4**	**5**	**6**
**1**	92064	722	0	0	0	0	0
**2**	24925	856	49	0	0	0	0
**3**	6388	428	107	24	0	0	0
**4**	1222	133	50	17	5	0	0
**5**	141	29	15	6	9	0	0
**6**	2	1	1	2	0	1	0

Supplementary Table 9: Influence of ABCG2, CYP2C9 and SLCO1B1 for statin-associated myopathy

Atorvastatin users with poor function ABCG2 vs with normal function ABCG2

	Univariate analysis				Multivariate analysis			
SAM	OR	Lower95%CI	Upper95%CI	Pvalue	OR	Lower95%CI	Upper95%CI	Pvalue
SAM	1.19	0.97	1.47	1.03E-01	1.20	0.97	1.49	8.51E-02
sSAM	1.90	1.11	3.25	1.94E-02	1.92	1.12	3.29	1.73E-02
myalgia	1.11	0.88	1.40	3.65E-01	1.12	0.89	1.42	3.24E-01
myositis	2.25	1.30	3.90	3.95E-03	2.27	1.31	3.94	3.58E-03

Fluvastatin users with decreased function ABCG2 vs with normal function ABCG2

	Univariate analysis				Multivariate analysis			
SAM	OR	Lower95%CI	Upper95%CI	Pvalue	OR	Lower95%CI	Upper95%CI	Pvalue
SAM	1.50	0.99	2.27	5.73E-02	1.50	0.99	2.27	5.64E-02
sSAM	0.43	0.15	1.20	1.08E-01	0.43	0.15	1.20	1.08E-01
myalgia	1.97	1.22	3.17	5.54E-03	1.97	1.22	3.18	5.39E-03
myositis	0.38	0.12	1.17	9.11E-02	0.38	0.12	1.17	9.00E-02

Simvastatin users with poor function ABCG2 vs with normal function ABCG2

	Univariate analysis				Multivariate analysis			
SAM	OR	Lower95%CI	Upper95%CI	Pvalue	OR	Lower95%CI	Upper95%CI	Pvalue
SAM	1.30	0.82	2.08	2.66E-01	1.34	0.84	2.13	2.22E-01
sSAM	3.53	1.48	8.40	4.34E-03	3.69	1.55	8.80	3.17E-03
myalgia	1.01	0.58	1.77	9.76E-01	1.03	0.59	1.81	9.10E-01
myositis	3.30	1.25	8.68	1.58E-02	3.43	1.30	9.05	1.26E-02
rhabdomyolysis	4.71	0.66	33.45	1.22E-01	5.03	0.71	35.77	1.07E-01

Atorvastatin users with decreased or poor function SLCO1B1 vs with normal function SLCO1B1

	Univariate analysis				Multivariate analysis			
SAM	OR	Lower95%CI	Upper95%CI	Pvalue	OR	Lower95%CI	Upper95%CI	Pvalue
SAM	1.26	1.09	1.46	2.03E-03	1.27	1.09	1.47	1.72E-03
sSAM	1.25	0.82	1.92	3.01E-01	1.26	0.82	1.93	2.98E-01
myalgia	1.26	1.08	1.48	3.94E-03	1.27	1.08	1.48	3.35E-03
myositis	1.29	0.81	2.08	2.86E-01	1.30	0.81	2.09	2.80E-01
rhabdomyolysis	1.11	0.41	3.01	8.39E-01	1.1	0.41	2.99	8.47E-01

Atorvastatin users with decreased function SLCO1B1 vs with normal function SLCO1B1

	Univariate analysis				Multivariate analysis			
SAM	OR	Lower95%CI	Upper95%CI	Pvalue	OR	Lower95%CI	Upper95%CI	Pvalue
SAM	1.23	1.05	1.43	8.22E-03	1.23	1.06	1.44	7.02E-03
sSAM	1.29	0.83	1.98	2.57E-01	1.29	0.83	1.99	2.54E-01
myalgia	1.22	1.03	1.43	1.78E-02	1.22	1.04	1.44	1.53E-02
myositis	1.32	0.81	2.13	2.64E-01	1.32	0.82	2.14	2.58E-01
rhabdomyolysis	1.18	0.43	3.2	7.46E-01	1.17	0.43	3.18	7.55E-01

Atorvastatin users with poor function SLCO1B1 vs with normal function SLCO1B1

	Univariate analysis				Multivariate analysis			
SAM	OR	Lower95%CI	Upper95%CI	Pvalue	OR	Lower95%CI	Upper95%CI	Pvalue
SAM	1.79	1.14	2.81	1.10E-02	1.78	1.13	2.78	1.23E-02
sSAM	0.75	0.10	5.40	7.76E-01	0.75	0.10	5.38	7.74E-01
myalgia	1.94	1.22	3.07	5.00E-03	1.92	1.21	3.04	5.63E-03
myositis	0.95	0.13	6.85	9.60E-01	0.95	0.13	6.84	9.58E-01

Simvastatin users with decreased or poor function SLCO1B1 vs with normal function SLCO1B1

	Univariate analysis				Multivariate analysis			
SAM	OR	Lower95%CI	Upper95%CI	Pvalue	OR	Lower95%CI	Upper95%CI	Pvalue
SAM	1.42	1.03	1.96	3.20E-02	1.42	1.03	1.96	3.22E-02

sSAM	2.91	1.43	5.91	3.15E-03	2.91	1.43	5.91	3.14E-03
myalgia	1.24	0.86	1.79	2.41E-01	1.24	0.86	1.79	2.41E-01
myositis	1.99	0.88	4.51	9.86E-02	1.99	0.88	4.52	9.83E-02
rhabdomyolysis	17.71	2.07	151.65	8.71E-03	17.72	2.07	151.75	8.70E-03

Simvastatin users with decreased function SLCO1B1 vs with normal function SLCO1B1

	Univariate analysis				Multivariate analysis			
SAM	OR	Lower95%CI	Upper95%CI	Pvalue	OR	Lower95%CI	Upper95%CI	Pvalue
SAM	1.41	1.01	1.96	4.38E-02	1.41	1.01	1.96	4.26E-02
sSAM	2.67	1.27	5.59	9.45E-03	2.68	1.28	5.61	9.17E-03
myalgia	1.23	0.85	1.79	2.80E-01	1.23	0.85	1.79	2.76E-01
myositis	1.89	0.81	4.43	1.41E-01	1.90	0.81	4.45	1.39E-01
rhabdomyolysis	15.14	1.69	135.53	1.51E-02	15.2	1.7	136.08	1.50E-02

Simvastatin users with poor function SLCO1B1 vs with normal function SLCO1B1

	Univariate analysis				Multivariate analysis			
SAM	OR	Lower95%CI	Upper95%CI	Pvalue	OR	Lower95%CI	Upper95%CI	Pvalue
SAM	1.66	0.61	4.54	3.24E-01	1.58	0.58	4.33	3.73E-01
sSAM	6.44	1.48	28.12	1.32E-02	5.88	1.34	25.78	1.89E-02
myalgia	1.45	0.46	4.61	5.30E-01	1.39	0.44	4.43	5.77E-01
myositis	3.44	0.45	26.11	2.32E-01	3.23	0.42	24.59	2.58E-01
rhabdomyolysis	55.06	3.43	884.17	4.66E-03	42.68	2.57	707.75	8.80E-03

Fluvastatin users with CYP2C9 LOF vs with CYP2C9 NM

	Univariate analysis				Multivariate analysis			
SAM	OR	Lower95%CI	Upper95%CI	Pvalue	OR	Lower95%CI	Upper95%CI	Pvalue
SAM	0.93	0.43	2.03	8.61E-01	0.93	0.43	2.03	8.63E-01
sSAM	1.32	0.31	5.71	7.07E-01	1.32	0.31	5.71	7.06E-01
myalgia	0.67	0.24	1.84	4.35E-01	0.67	0.24	1.84	4.37E-01
myositis	1.47	0.34	6.41	6.05E-01	1.47	0.34	6.40	6.06E-01

Fluvastatin users with CYP2C9 IM vs with CYP2C9 NM

	Univariate analysis				Multivariate analysis			
SAM	OR	Lower95%CI	Upper95%CI	Pvalue	OR	Lower95%CI	Upper95%CI	Pvalue
SAM	0.82	0.36	1.89	6.44E-01	0.82	0.36	1.89	6.45E-01
sSAM	1.36	0.32	5.88	6.78E-01	1.36	0.32	5.88	6.78E-01
myalgia	0.51	0.16	1.64	2.62E-01	0.52	0.16	1.65	2.63E-01
myositis	1.51	0.35	6.58	5.80E-01	1.51	0.35	6.58	5.81E-01

Fluvastatin users with CYP2C9 PM vs with CYP2C9 NM

	Univariate analysis				Multivariate analysis			
SAM	OR	Lower95%CI	Upper95%CI	Pvalue	OR	Lower95%CI	Upper95%CI	Pvalue
SAM	0.93	0.43	2.03	8.61E-01	0.93	0.43	2.03	8.63E-01
myalgia	0.67	0.24	1.84	4.35E-01	0.67	0.24	1.84	4.37E-01

Supplementary Table 10: Patient characteristics of NSAID users in TPMI

NSAID Users	no ADE		ADE		OR (95% CI)	P value
No. of subjects	9471		529			
Male	3288	34.7%	205	38.8%	1.19 (0.99-1.42)	6.00E-02
Age (years, mean±SD)	61±14		61.8±14.3			1.10E-01
Dose_first (DDD)	1.1±0.84		1.22±0.4			6.00E-02
Comorbidity						
HTN	1857	19.6%	213	40.3%	2.76 (2.3-3.31)	3.62E-30
HC	1940	20.5%	178	33.6%	1.97 (1.63-2.38)	5.51E-13
DM	1466	15.5%	159	30.1%	2.35 (1.94-2.85)	9.15E-19
CVD	500	5.3%	65	12.3%	2.51 (1.91-3.3)	1.09E-11
IHD	817	8.6%	80	15.1%	1.89 (1.47-2.42)	3.60E-07
PVD	23	0.2%	11	2.1%	8.72 (4.23-17.99)	1.64E-12
COPD	43	0.5%	14	2.6%	5.96 (3.24-10.96)	7.09E-11
HF	122	1.3%	35	6.6%	5.43 (3.69-7.99)	8.50E-22
HP	6	0.1%	0	0.0%		5.63E-01
Concurrent medications						
aspirin	769	8.1%	88	16.6%	2.26 (1.78-2.87)	9.80E-12
antibiotics	3	0.0%	5	0.9%	30.11 (7.18-126.34)	4.76E-13
ACEI/ARB	20	0.2%	9	1.7%	8.18 (3.71-18.05)	5.55E-10
diuretics	66	0.7%	80	15.1%	25.39 (18.08-35.65)	1.25E-159
anticoagulants	45	0.5%	5	0.9%	2 (0.79-5.06)	1.36E-01
immunosuppressants	36	0.4%	11	2.1%	5.57 (2.82-11.01)	2.68E-08
CYP2C9						
EM	8757	92.5%	499	94.3%		
IM	693	7.3%	28	5.3%	0.71 (0.48-1.05)	8.13E-02
PM	12	0.1%	2	0.4%	2.92 (0.65-13.08)	1.41E-01
Indetermined	9	0.1%	0	0.0%		4.74E-01

Supplementary Table 11: Influence of ABCG2, CYP2C9 and SLCO1B1 for statin-associated myopathy

NSAID users with decreased or poor function CYP2C9 vs with normal function CYP2C9

	Univariate analysis				Multivariate analysis			
ADE	OR	Lower95%CI	Upper95%CI	Pvalue	OR	Lower95%CI	Upper95%CI	Pvalue
ADE	0.75	0.51	1.09	0.13	0.75	0.51	1.1	0.14
GI	0.8	0.48	1.34	0.41	0.8	0.48	1.34	0.40
kidney	0.72	0.49	1.06	0.09	0.72	0.49	1.07	0.10

NSAID users with decreased function CYP2C9 vs with normal function CYP2C9

	Univariate analysis				Multivariate analysis			
ADE	OR	Lower95%CI	Upper95%CI	Pvalue	OR	Lower95%CI	Upper95%CI	Pvalue
ADE	0.71	0.48	1.05	0.08	0.71	0.47	1.05	0.09
GI	0.72	0.42	1.23	0.23	0.71	0.41	1.23	0.23
kidney	0.71	0.48	1.05	0.08	0.71	0.47	1.05	0.09

NSAID users with poor function CYP2C9 vs with normal function CYP2C9

	Univariate analysis				Multivariate analysis			
ADE	OR	Lower95%CI	Upper95%CI	Pvalue	OR	Lower95%CI	Upper95%CI	Pvalue
ADE	2.92	0.65	13.1	0.16	3.37	0.75	15.14	0.11
GI	6.03	1.34	27.08	0.02	5.97	1.33	26.87	0.02
kidney	1.35	0.18	10.34	0.77	1.56	0.2	11.93	0.67

Supplementary Table 12: Descriptions of NHIRD order codes for dialysis and renal replacement therapy procedures

NHIRD Order Code	Name
58001C	Hemodialysis
58002C	Peritoneal dialysis
58009B	Continuous ambulatory peritoneal dialysis， CAPD - 1.CAPD instruction
58010B	Continuous ambulatory peritoneal dialysis, CAPD - 2.CAPD, single unit P.D. set transfer material fee
58011C	Continuous ambulatory peritoneal dialysis, CAPD 3.Peritoneal Dialysis Follow up therapy (1)CAPD
58012B	Continuous ambulatory peritoneal dialysis， CAPD 4.CAPD，Tenckhoff catheter implantation
58013C	Ascites dialytic ultrafiltration
58017C	Continuous ambulatory peritoneal dialysis, CAPD 3.Peritoneal Dialysis Follow up therapy (2)Automated peritoneal dialysis
58018C	Continuous veno-venous hemofiltration dialysis (C.V.V.H.D)
58026C	Home visit - home dialysis therapy
76020B	Renal implantation
N26028	Da Vinci assisted renal implantation

Figure s1: study design for clopidogrel-related adverse drug reactions

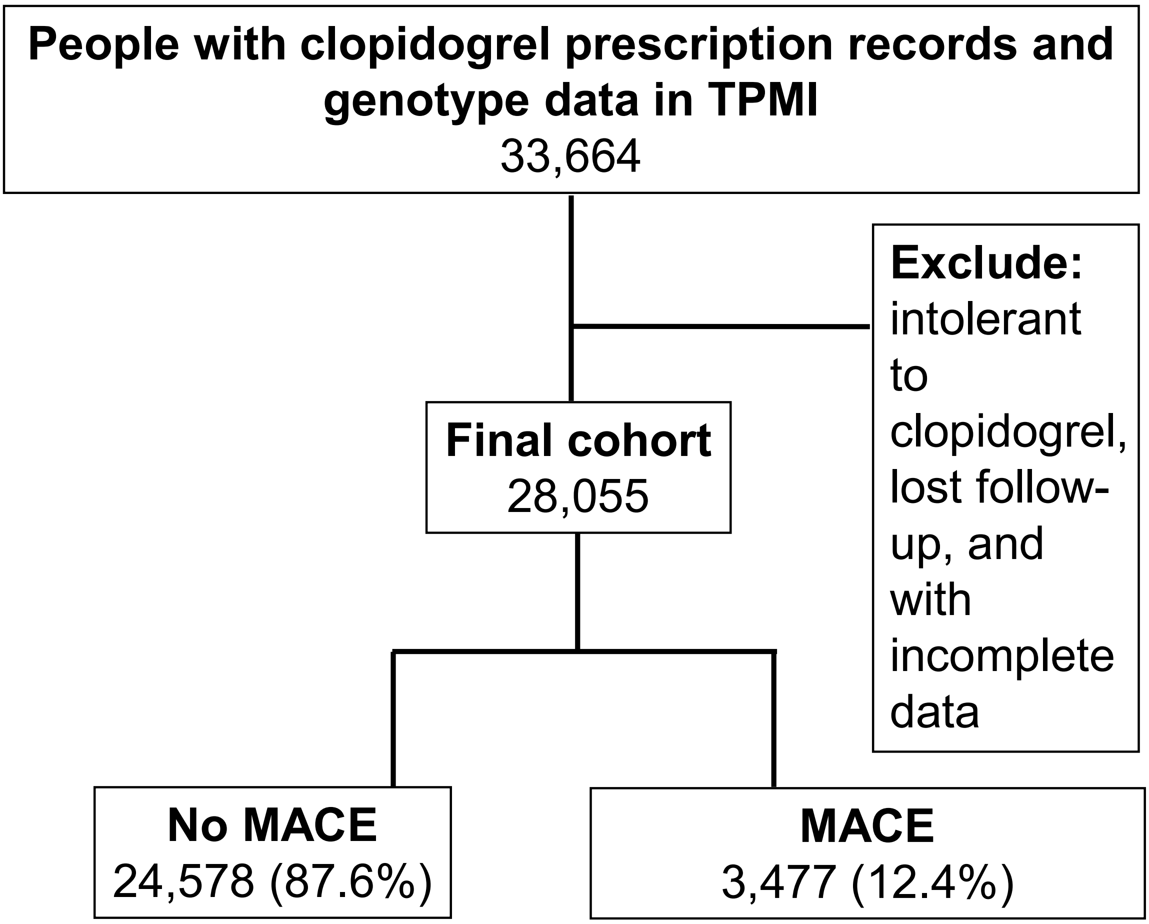

Figure s2: study design for azathioprine-related adverse drug reactions

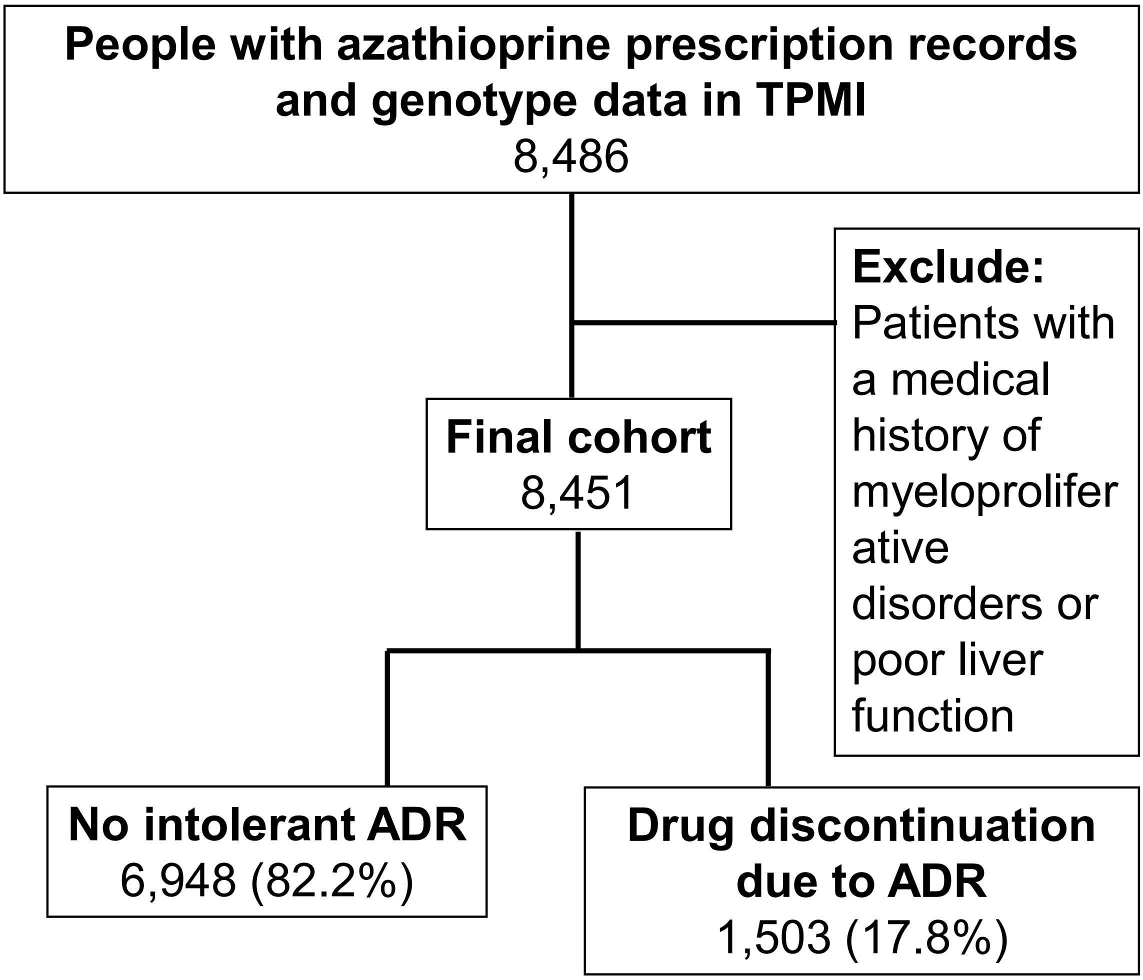

Supplement: Supplementary file 1 — Supplementary Information [file 41467_2025_61644_MOESM1_ESM.pdf]
